# Role of Brushing and Occlusal Forces in Non-Carious Cervical Lesions (NCCL)

**Published:** 2014-12

**Authors:** Durre Sadaf, Zubair Ahmad

**Affiliations:** Dental College, Qassim University, Qassim, Saudi Arabia

**Keywords:** Non-carious cervical lesions (NCCL), occlusal facets, Teeth sensitivity

## Abstract

**Objective:**

To assess the association of occlusal forces and brushing with non-carious cervical lesions (NCCL).

**Methodology:**

It was a Cross-sectional study. The study was conducted in Dental clinics, Department of Surgery, The Aga Khan University Hospital Karachi. The study duration was from 1^st^ January 2009 to 28^th^ Feb 2009. Ninety patients visiting dental clinic were examined clinically. Presence of Non- carious cervical lesions, broken restorations, fractured cusps, presence of occlusal facets, brushing habits, Para functional habits were assessed. All the relevant information and clinical examination were collected on a structured Performa and was analyzed using SPSS version 14.0. . Chi square χ^2^ test was applied to assess association among different categorical variables.

**Result:**

Twenty three (26%) females and 67 (74%) males were included in the study. Thirty five of them (38.9%) were found to have Non-carious cervical lesions. Presence of NCCL has no association with gender (*P* value 0.458). A significant association was found between NCCL and teeth sensitivity (*P* value 0.002).The association between use of hard tooth brush and Non-carious cervical lesions was found significant (*P* value <0.001). However the association among Non-carious cervical lesions and fractured cups, broken restoration, teeth grinding, jaw clenching, pan chalia chewing and frequency of teeth brushing were insignificant.

**Conclusion:**

Hard tooth brushing and teeth sensitivity have significant association with Non-carious cervical lesions. The role of occlusal wear in the formation of NCCL is not significant.

## INTRODUCTION

The factors that are responsible for loss of tooth structure other than caries at cervical areas of the teeth are complex and are not fully understood (1). Loss of tooth structure at cervical area of teeth may be responsible for teeth sensitivity and esthetic problems (1). Early studies suggested that the tooth loss was associated with brushing (abrasion) and acidic drinks (erosion) (1, 2). This concept did not fully explain the reason for loss of tooth structure at cervical area involving single tooth, subgingival loss of tooth structure and wedge shaped lesions (1). In 1991, Grippo introduced a new category called “abfraction” which he referred to the pathological loss of dental tissues caused by biomechanical forces (3). He suggested that predominant factor responsible for NCCL was occlusal forces. Tooth flexure leads to enamel and dentin fatigue.

Tooth flexure is the lateral or an axial bending of tooth under occlusal forces. According to tooth flexure theory, occlusal forces are transmitted through axial surface to cervical areas. These forces generate tensile stresses. This results in disruption of chemical bond between dentine and enamel hydroxyapatite crystals resulting in tooth surface loss at cervical areas (4).

However there is little evidence supporting abfraction as the primary factor (5). Understanding the etiology and mechanism involved in cervical tooth wear may affect restorative techniques. Restorative failure may be related to the tooth flexure and stresses at the cervical areas, since some forces acting on the enamel and dentin at the cemento-enamel junction may cause the restorative material to undergo debonding, marginal leakage and loss of retention (5). The need for conducting such studies would greatly help clinicians to formulate preventive and restorative treatment modalities.

It may help us to determine the optimum properties of materials used to restore Class V cervical lesions including those materials that can withstand tooth brush abrasion or resist the tensile forces at cervical areas. Knowledge about the etiology may provide the possibility of early diagnosis and prevention of NCCL (6).

The purpose of this study is to clinically assess the frequency of NCCL and determine the role of occlusal forces and tooth brushing in tooth loss at cervical area.

## METHODS AND MATERIALS

It was a Cross-sectional study. The study was conducted in Dental clinics, Department of Surgery, The Aga Khan University Hospital Karachi. The study duration was from 1^st^ January 2009 to 28^th^ Feb 2009. Clearance was obtained from Ethical Review Committee of the University. Ninety patients visiting dental clinic were examined. Presence of NCCL, broken restorations adjacent to NCCL , fractured cusps adjacent to NCCL, presence of occlusal facets adjacent to NCCL, brushing habits, presence of teeth sensitivity adjacent to NCCL, habits of teeth grinding, jaw clenching and pan chalia chewing were assessed. All the relevant information and clinical examination were collected on a structured Performa and was analyzed using SPSS version 14. Chi square χ^2^ test was applied to assess association among different categorical variables.

## RESULTS

Twenty three (26%) females and 67 (74%) males were included in the study (Table 1). Thirty five of them (38.9%) were found to have NCCL. Gender difference regarding presence of NCCL was not significant (*P* value 0.458) (Table 2).

**Table 1:** Frequencies and Percentages of Variables

Variables		% age (n)	Total % age (n)

Gender	% Male (n)	74.4% (67)	100% (90)
	% Female (n)	25.6% (23)	
NCCL	% Present (n)	38.9% (35)	100% (90)
	% Absent (n)	61.1% (55)	
Fractured cusps	% Present (n)	14.4% (13	100% (90)
	% Absent (n)	85.6% (77)	
Broken restoration	% Present (n)	7.8% (7)	100% (90)
	% Absent (n)	92.2% (83)	
Teeth sensitivity	% Present (n)	22% (20)	100% (90)
	% Absent (n)	77.8% (70)	
Brushing frequency	% Two Times (n)	98.9% (89)	100% (90)
	% More than two (n)	1.1% (1)	
Brush Type	% Soft/Medium (n)	72.2% (65)	100% (90)
	% Hard (n)	27.8% (25)	
Flat cusps/cusp facets	% Present (n)	53.3% (48)	100% (90)
	% Absent (n)	46.7% (42)	

**Table 2:** Association between NCCL and Gender, tooth sensitivity, fractured cusp, brushing frequency, tooth brush type and flat cusps/cusps facets (Chi Square Test)

Variables		NCCL		*P* value
		Present	Absent	

Gender	Male	28	39	0.458
	Female	7	16	
Tooth sensitivity	Present	14	21	0.002
	Absent	6	49	
Fractured cusp	Present	4	31	0.759
	Absent	9	46	
Brushing frequency	Twice daily	34	1	0.389
	Thrice daily	55	1	
Type of tooth brush	Soft/Moderate	12	23	<0.001
	Hard	53	2	
Flat cusps / Cusp facets	Present	20	15	0.666
	Absent	28	27	
Jaw clenching	Present	3	2	
	Absent	32	53	0.294
Pan chalia chewing	Present	3	7	
	Absent	32	48	0.403
Teeth grinding	Present	2	1	
	Absent	33	54	0.334

A significant association was found between presence of NCCL and teeth sensitivity (*P* value 0.002) (Table 2).

The association between use of hard tooth brush and NCCL was found significant (*P* value <0.001).

However the association among NCCL and fractured cups, broken restoration, teeth grinding, jaw clenching, pan chalia chewing and frequency of teeth brushing was not significant (Table 2). Use of carbonated drinks is not found be significantly associated with NCCL. Occlusal wear in the form of flat cusps and cusps facets is not associated with NCCL significantly (*P* value 0.666).

## DISCUSSION

A significant association was found between NCCL and teeth sensitivity in this study. It is supported by a study done on Swiss adults in which 84.6% patients with NCCL presented with hypersensitivity (7). These results were in contrast to Hina (8) who found no sensitivity in 71% of patients with NCCL present. The reason for teeth sensitivity is due to enamel loss at cervical area leading to dentin exposure. However long standing NCCL gradually may become less sensitive due to formation of reparative or sclerotic dentine.

Use of hard tooth brush and excessive brushing has been found to be associated with NCCL significantly. Our results are similar to John J. Dzakovich (9) who worked on relationship between abrasion and NCCL. His results showed that horizontal brushing method with tooth pastes was capable of tooth loss at cervical areas (9). Another study showed greater frequency of NCCL in patients who brushed twice daily, compared with those who brushed less frequently (10, 11). However, frequency of brushing was not found to be associated with NCCL significantly in this study. In another study use of hard tooth brush is incapable of abrading enamel but capable of producing surface roughness in dentine, it indirectly supports the concept that the abrasion and erosion have a secondary role in causing NCCL (12). Another clinical study showed tooth brush abrasion is strongly suspected as contributing to the formation of the majority of wedge shaped lesions, whereas the presence or contribution of occlusal stresses in the direct formation of these lesions could not be measured directly (13).

A laboratory investigation done by Davis and Winter (14) introduced the concept of combined role of abrasion and erosion. According to this concept, the primary factors are the alternate tensile and compressive stresses that cause theoretical disruption of the bond between enamel rods and increase the enamel susceptibility to dissolution and abrasion (15).

Association between gender and cervical wear was not found significant. Similar results are discussed in a critical review on NCCL done by Bartlett (10) that showed that gender difference on prevalence of cervical wear was not found in various studies (14-17). In this study, the association between NCCL and gender was also found insignificant.

In our study parafunctional habits like teeth grinding has no association with cervical wear, similar finding done by Luiz Fernando (1).

Much of the evidence for abfraction has been derived from finite element studies and few early engineering models (18). Finite element studies are based on computerized procedures, a series of theoretical load is applied on a model, and its impact is assessed along the length of the model (19-20). These studies were based on the hypothesis that continual occlusal loading produces tensile stresses that lead to initiation of crack. However a contradictory evidence from the finite element studies suggests that the lingual walls of the teeth should be equally as susceptible to cervical wear as are the buccal walls but this is not supported by the clinical findings, where lingual lesions are rare (5). Eccentric forces and heavy occlusal loadings were not significantly associated with tooth surface loss at cervical area in our study Experimental and clinical evidence is required to show if and how this phenomenon occurs (1).

Heavy Occlusal forces in the form of the broken restorations, cusp fracture and pan chalia chewing were not significantly associated with cervical wear.

David and Winter reported the role of erosion and abrasion in NCCL (14). That is only one clinical study that has investigated the progression of cervical wear lesion affected by dietary acids and frequency of tooth brushing. However in this study, acidic drinks are not found to be associated with cervical wear significantly.

Recognizing and understanding the etiology of NCCL may affect on prevention and management of such lesions.

Restoration at cervical region of the tooth is a big challenge for a clinician. Occlusal evaluation before restoration is essential (6). Eccentric forces on the teeth produce lateral flexure that produces tensile stresses at marginal interface of the restoration. These stresses could be responsible for adhesive failure of cervical composite restoration or may result in microleakage or partial debonding of the restoration (20).

## CONCLUSIONS

Occlusal forces and occlusal trauma is not proven clinically responsible for production NCCL as primary factor. However, NCCL may develop as a result of interaction of multiple factors. Excessive brushing and use of hard tooth brush have strong associations with cervical tooth wear. There is need to educate the patients about proper bushing techniques.
